# Identification and functional prediction of long non-coding RNA and mRNA related to connective tissue disease-associated interstitial lung diseases

**DOI:** 10.2478/rir-2023-0030

**Published:** 2023-12-19

**Authors:** Fei Dai, Yixi He, Tianyi Lei, Yi Jiang, Quanbo Zhang, Yufeng Qing

**Affiliations:** Research Center of Hyperuricemia and Gout, the Afiliated Hospital of North Sichuan Medical College, Nanchong 637000, Sichuan Province, China; Department of Rheumatology and Immunology, the Affiliated Hospital of North Sichuan Medical College, Nanchong 637000, Sichuan Province, China; Department of Geriatrics, the Afiliated Hospital of North Sichuan Medical College, Nanchong 637000, Sichuan Province, China

**Keywords:** connective tissue disease-associated interstitial lung disease, long non-coding RNA, microarray analysis, functional analysis

## Abstract

**Objective:**

Recently, the role of long non-coding RNA (lncRNA) in rheumatic immune diseases has attracted widespread attention. However, knowledge of lncRNA in connective tissue disease-associated interstitial lung disease (CTD-ILD) is limited. This study explored the expression profile and possible mechanisms of lncRNA and mRNA in peripheral blood mononuclear cells (PBMCs) of CTD-ILD patients, especially systemic sclerosis (SSc)-ILD and rheumatoid arthritis (RA)-ILD.

**Methods:**

LncRNA microarray analysis identified 240 diferentially expressed lncRNAs and 218 diferentially expressed mRNA in the CTD-ILD group and the connective tissue disease without associated interstitial lung disease (CTD-NILD) group. The bioinformatics analysis of diferential genes has identified several important biological processes and signal pathways, including nuclear factor kappa B (NF-kappa B) signaling pathway, interleukin 17 (IL-17) signaling pathway, B cell receptor signaling pathway. Relative expression levels of five diferentially expressed lncRNAs and one mRNA in 120 SSc and RA patients with or without ILD were detected by quantitative reverse-transcription (PCR).

**Results:**

The *ENST00000604692* expression level was significantly higher in the ILD than the without interstitial lung disease (NILD) group; *T311354* and arginase-1 were significantly higher in SSc than RA group.

**Conclusion:**

These data suggest that the specific profile of lncRNA in PBMCs of CTD-ILD patients and the potential signal pathways related to the pathogenesis of CTD-ILD, which may provide newfound insights for the diagnosis and treatment of CTD-ILD patients.

## Introduction

Connective tissue disease (CTD) is an autoimmune disease characterized by chronic inflammation of blood vessels and connective tissue.^[1]^ The lung is a vital organ with distinct functions like secretion, metabolism, and immune defence. As the lung is rich in connective tissues such as collagen and blood vessels, CTD frequently involves the lung.^[1,2]^ Interstitial lung disease (ILD) is the most common manifestation of diffuse disease that occurs in the lung interstitium (including bronchi, perivascular, lobular septum, and alveolar septum). CTD-associated ILD (CTD-ILD) poses a serious threat to the lives and health of patients and is one of the main causes of death in the acute phase of CTD.^[3]^ ILD can present in multiple types of CTD，such as systemic sclerosis (SSc), rheumatoid arthritis (RA), Sjogren’s syndrome (SS), systemic lupus erythematosus (SLE), polymyositis/dermatomyositis (PM/DM), and so on.^[1]^ The incidence of various CTD-ILD reported in the literature is about: 40% to 91% in SSc, 6.5% to 33% in RA, 1% to 15% in SLE, 9% to 20% in SS, and 19.9% to 86% in PM/DM.^[3]^ The relevant literature review has shown that the mortality rate of some CTD-ILD is about 20% in RA, and the 10-year mortality rate in SSc is as high as 40%.^[3]^ Although genetics, smoking, air pollution, gastroesophageal reflux, occupational exposure, viral infection, aging and other factors have been confirmed to have important effects on CTD-ILD, the specific pathogenesis of it has not yet been fully elucidated.^[1, 2, 3]^ CTD-ILD patients have no apparent symptoms in the early stage, and irreversible pulmonary interstitial fibrosis often occurs in the late stage, leading to multiple complications, including sleep-disordered breathing, dyspnea, and cough. Thus, elucidating the exact pathogenesis of CTD-ILD is particularly important for guiding early clinical diagnosis and treatment.

Long non-coding RNA (lncRNA) is a single-stranded RNA molecule with a length greater than 200 nt that lacks the ability to encode proteins.^[4]^ The wide subcellular distribution of lncRNA in cells determines the diversity of their functional mechanisms. For example, lncRNA in the nucleus can serve as molecular scaffolds, assist alternative splicing, and regulate chromosome structure, while lncRNA in the cytoplasm can regulate translation, promote or inhibit mRNA degradation, adsorption of miRNA, *etc*.^[5, 6, 7]^ More importantly, aberrant lncRNA expression has been associated with various kinds of human diseases, such as central nervous system disease,^[8]^ cardiovascular diseases,^[9]^ cancer,^[10]^ autoimmune diseases.^[11]^ Detecting the expression of lncRNA in different cells or disease states can therefore contribute to better understanding its function, reveal its mechanism of action, or identify effective biomarkers.

Although there have been many studies related to lncRNA, investigations regarding their functional roles in CTD-ILD remain limited. The study analyzed the differentially expressed lncRNA and mRNA in peripheral blood mononuclear cells (PBMCs) of CTD-ILD patients, and provided new insights into the pathogenesis of CTD-ILD from the genetic level.

## Materials and Methods

### Subject Selection

All participants were recruited from the Department of Rheumatology of the Affiliated Hospital of North Sichuan Medical College between 2020 and 2022. They all fulfilled the diagnostic criteria for CTD or CTD-ILD: SSc, RA, SLE, and SS patients fulfilled the revised American College of Rheumatology/European League Against Rheumatism diagnostic criteria.^[12, 13, 14, 15]^ All diagnoses of ILD were made following the 2013 American Thoracic Society/European Respiratory Society guideline.^[16]^ Exclusion criteria: (1) patients with underlying lung diseases, such as lung tumors, tuberculosis, and other infections in the lungs; (2) patients with ILD caused by environmental, occupational, drug, infection, and other factors; (3) patients with severe organ dysfunction such as heart, liver, and kidney; (4) patients with active chronic or acute infectious diseases.

The clinical data of the subjects, including age, sex, high-resolution chest computed tomography (CT) findings，laboratory results were collected. All high-resolution chest CT examinations and laboratory indicators were carried out by the Imaging Department and Clinical Laboratory Department of the Affiliated Hospital of North Sichuan Medical College respectively. The basic information of all research subjects was shown in Supplementary Table S1. This study was approved by the Ethics Committee of the Affiliated Hospital of North Sichuan Medical College (the approval number is 2022ER280-1) and was conducted in accordance with the ethical guidelines of the 1975 Declaration of Helsinki. Part of the design, analysis and interpretation of the present study are based on previous reports.^[17]^

### Study Design

The study was designed in two steps: a discovery phase and a validation phase. In the first stage, total RNA was isolated from PBMCs of 4 SSc-ILD, 4 RA-ILD, 4 SLE-ILD, and 4 SS-ILD, respectively. To reduce the uncertain factors caused by a single sample, all 16 ILD samples were evenly mixed to obtain four RNA specimens of CTD-ILD, that is, each specimen was obtained by equal mixing of RNA from 1 case each of SSc-ILD, RA-ILD, SLE-ILD and SS-ILD randomly selected. Similarly, 3 SSc-without ILD (NILD), 3 RA-NILD, 3 SLE-NILD, and 3 SS-NILD were mixed to obtain 3 CTD-NILD RNA specimens. CTD-ILD group was paired with age- and sex-matched persistently CTD-NILD group (Supplementary Table S2). Arraystar Human LncRNA Microarray V4.0 (Arraystar, Rockville, MD, USA) was used to detect the expression profiles of lncRNA and mRNA in 4 CTD-ILD and 3 CTD-NILD specimens. In this study, samples from different CTD-associated ILD were mixed for high-throughput sequencing. First of all, these samples were from patients with CTD. CTD includes a variety of diseases with different clinical manifestations. However, the number of samples used for microarray sequencing was small. In order to make the research more meaningful, we selected several common CTDs, including SSc, RA, SLE, SS. Secondly, we divided CTD into ILD group and NILD group, and mixed samples from different CTD patients to reduce the bias between individuals. In the second phase, 60 ILD patients (30 SSc-ILD, 30 RA-ILD) and 60 sex- and age-matched NILD patients (30 SSc-NILD, 30 RA-NILD) were included in the study, and qRT-PCR was used to determine the relative quantitative expression of selected genes. Previous studies suggest that the incidence rate of ILD was higher in SSc and RA.^[3]^ Meanwile, among our outpatient and inpatient patients, SSc-ILD and RA-ILD are the most commonly observed CTD-ILD, which also facilitates us to collect more research objects. So, in the second stage of this study, only PBMC samples from RA and SSc associated with ILD patients were selected for real-time quantitative PCR validation.

### Sample Preparation and RNA Extraction

The heparin anticoagulant tube was used to collect 4 mL fasting peripheral blood from all subjects. Separate PBMCs by Ficoll density gradient centrifugation and transfer them to fresh RNase-free test tubes. The total RNA from PBMCs was extracted according to the instructions of Trizol kit (Invitrogen, Carlsbad, CA, USA). The integrity of RNA was evaluated by standard denaturing agarose gel electrophoresis. The concentration, purity, and integrity of RNA were tested using NanoDrop ND-1000 spectrophotometer (NanoDrop Technologies, Wilmington, DE, USA) and an Agilent 2100 Bioanalyzer (Agilent Technologies, Inc., Santa Clara, CA, USA).

### Microarray

Arraystar Human lncRNA Microarray V4.0 was adopted for the detection of lncRNA and mRNA expression profiles in the CTD-ILD group and CTD-NILD group. Sample labeling and chip hybridization were performed according to the Agilent One-Color Microarray-Based Gene Expression Analysis protocol (Agilent Technologies, Inc., Santa Clara, CA, USA). All microarray work was performed by Kangcheng Bio-tech Inc (Shanghai, China). After hybridization, washing, and staining, the microarray chips were scanned using the Agilent DNA Microarray Scanner G2505C (Agilent Technologies, Inc., Santa Clara, CA, USA) to get the microarray results.

### Data Analysis and Diferentially Expressed incRNA/mRNA Identification

Agilent Feature Extraction software (version 11.0.1.1) was used to analyze the acquired array images. Quantile normalization and subsequent data processing were performed using the R (version 3.6.1) limma package.

### Bioinformatics Analysis

The diferential expression genes with a fold change > 2 were selected for the Gene Ontology (GO)（http://www.geneontology.org）analysis and Kyoto Encyclopedia of Genes and Genomes (KEGG) (http://www.genome.jp/kegg/) pathway analysis to investigate their biological functions and related signal transduction pathways.

### qRT-PCR

Based on the microarray results, the diferentially expressed target lncRNA and mRNA were selected and examined by qRT-PCR. According to the instructions of the reverse transcription kit (Takara, Kyoto, Japan), 2 μL (600 ng) of total RNA was reverse transcribed into cDNA. Then, qRT-PCR was carried out on the LightCycle®96 PCR machine (Roche, Basel, Switzerland). All reactions were performed in triplicate. In this study, β-actin was used as an internal reference gene. The primer sequences of all genes used for qRT-PCR (Shanghai Shenggong Bioengineering Co., Ltd., Beijing, China) were listed in Supplementary Table S3.

### Statistical Analysis

IBM SPSS Statistics 23.0 and GraphPad Prism 8.0 statistical software were used for analysis. Quantitative data of approximately normal distribution were described by the mean ± standard deviation, and non-normal distribution data were described by the median and interquartile range (IQR). Comparisons between groups were performed using Student’s *t*-test or the Mann-Whitney *U* test. Correlations were calculated using Spearman’s rank correlation test. A receiver operating characteristic (ROC) curve was used to evaluate the diagnostic eficacy of the candidate biomarker. A *P* value less than 0.05 was considered statistically significant and all tests were two-sided.

## Results

### LncRNA and mRNA Expression Profiles

**Figure 1:**
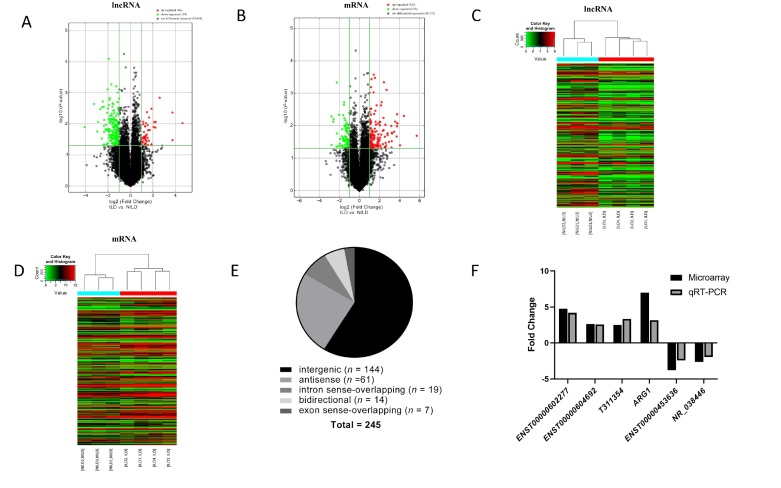
Expression profiles of lncRNA and mRNA in connective tissue disease-associated interstitial lung diseases (CTD-ILD, n = 4) group compared with connective tissue disease-without interstitial lung diseases (CTD-NILD, n = 3) group. (A, B) Volcano plots visualizing differentially expressed lncRNAs and mRNAs between the two groups. The vertical lines correspond to 2-fold up- and downregulated genes expression. The horizontal line represents a P-value of 0.05. The red point in the plot represents significantly differentially expressed genes. (C, D) Clustered heatmap showing the comparison of all important lncRNAs and mRNAs between the two groups. Expression values are represented by the color scale. The intensity increases from green (relatively low expression) to red (relatively high expression). Each column represents one sample, and each row represents a single lncRNA or mRNA. (E) LncRNA classification and subgroup analysis. (F) Relative expression levels of the five lncRNAs and one mRNA in the two groups. The Y-axis represents the ratio of the relative expression level of genes in the CTD-ILD group compared with CTD-NILD group.

Microarray data have been uploaded to GEO accession number GSE192985: https://www.ncbi.nlm.nih.gov/geo/query/acc.cgi?acc=GSE192985. Volcano Plot and Hierarchical Clustering (Figure 1A-D) showed the diferences in lncRNA and mRNA expression between the two groups. Based on microarray analysis (fold change > 2, *P* < 0.05), compared with the CTD-NILD group, the CTD-ILD group had a total of 46 lncRNAs and 133 mRNAs up-regulated, 194 lncRNAs and 85 mRNAs down-regulated. Gene expression is a complex and highly regulated process, and lncRNA acts as a basic regulator of gene expression at the epigenetic level. According to the position of lncRNA in the genome and the relationship to its nearest protein-coding gene, lncRNA can be classified into the following types: intergenic, natural antisense, intronic antisense, bidirectional, intron sense-overlapping, exon sense-overlapping.^[18]^ For further investigation of potential function of diferential genes, lncRNA classification and subgroup analysis were conducted (Figure 1E). Among them, intergenic lncRNA accounted for the largest proportion (58.78%, followed by antisense lncRNA (24.90%). Additionally, intergenic lncRNA, also called lincRNA, is mainly produced in the middle region of two coding genes.^[19]^ Recent studies have shown that lincRNA is one of the focuses in biomedical research, and many lincRNAs have gradually been discovered to be key regulators of various biological processes.^[20]^ For example, enhancer-like lncRNA is also a type of lincRNA, located in the enhancer region of the genome, which can activate the transcription of adjacent genes.^[21]^ A coexpression analysis was used to further determine the potential functions of diferentially expressed lncRNA subgroup in CTD-ILD. A total of ten pairs of lincRNA-mRNA (Supplementary Table S4) and two pairs of antisense lncRNA-mRNA (distance < 300 kb) (Supplementary Table S5) were indicated to be coregulated transcripts.

### qRT-PCR Validation

To determine the validity of the microarray results, four differentially expressed lncRNA identified by the microarray were randomly selected for qRT-PCR analyses. In addition, to obtain deeper insights into the functions of lincRNA in CTD-ILD，diferential lincRNAs were selected for conjoint analysis with neighboring coding genes (< 300 kb). Among them, arginase-1 (*ARG1*) is the neighboring differential gene of *T311354*, which was selected together for qRT-PCR analysis in this study. The detailed information of all five lncRNAs (*ENST00000602277*, *ENST00000604692*, *ENST00000453636*, *NR_038446*, *T311354*) and one mRNA (*ARG1*) was shown in Supplementary Table S6. To date, nothing has been published about the functions of these genes in CTD-ILD. The expression levels of five target lncRNAs and one mRNA in 7 specimens of the previous microarray analysis were detected by qRT-PCR. As shown in Figure 1F，the trend was consistent with the microarray data, indicating that the microarray results were reliable.

### GO and KEGG Pathway Analyses of Diferentially Expressed mRNAs

**Figure 2:**
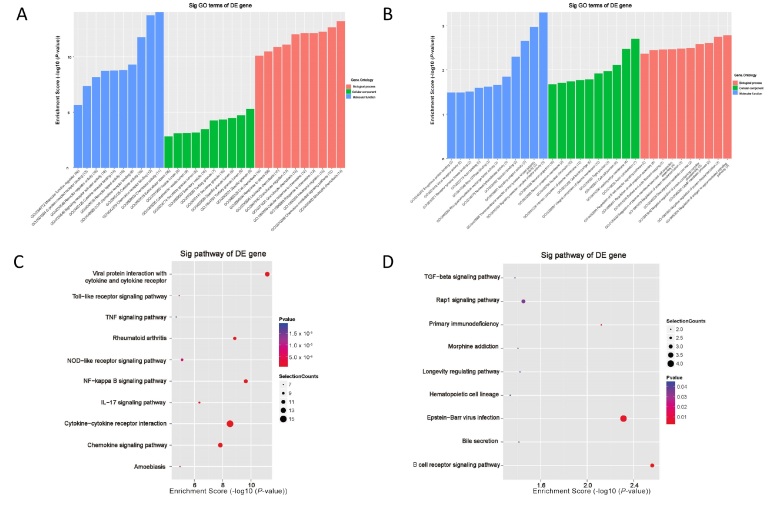
Bioinformatics analysis. (A, B) Gene Ontology (GO) analysis enriches the top 10 items in the molecular function, cell composition, and biological process of upregulated and downregulated mRNAs. (C, D) Kyoto encyclopedia of genes and genomes (KEGG) analysis enriched the top 10 items of upregulated mRNAs and the top 9 items (only 9 items in total) of downregulated mRNAs. TNF, tumor necrosis factor; NOD, nucleotide oligomerization domain; NF-kappa B, nuclear factor kappa B; IL-17, interleukin 17; TGF-β, transforming growth factor-β.

GO analysis was performed to assess the biological processes (BP), cell components (CC), and molecular functions (MF) involved in the diferential genes. According to the results of GO enrichment analysis, the main enriched important terms were related to immune-inflammatory system processes, such as “neutrophil chemotaxis (GO:0030593)”, “chemokine-mediated signaling pathway (GO:0070098)”, “regulation of antigen receptor-mediated signaling pathway (GO:0050854)”, “regulation of B cell receptor signaling pathway (GO:0050855)”, “signaling receptor complex adaptor activity (GO:0030159)”, “signaling adaptor activity (GO:0035591)”. The top 10 significantly enriched GO terms in the BP, CC, and MF categories are shown in Figure 2A, 2B (Supplementary Table S7), respectively.

The KEGG database was used to investigate the enriched pathways. A total of 33 pathways related to upregulated mRNAs and 9 pathways related to downregulated mRNAs were identified, including “nuclear factor kappa B (NF-kappa B) signaling pathway (hsa04064)”, “interleukin 17 (IL-17) signaling pathway (hsa04657)”, “nucleotide oligomerization domain (NOD)-like receptor signaling pathway (hsa04621)”, “Toll-like receptor signaling pathway (hsa04620)”, “tumor necrosis factor (TNF) signaling pathway (hsa04668)”, “B cell receptor signaling pathway (hsa04662)”, *etc*. The most enriched pathways of upregulated and downregulated diferential genes were shown in Figure 2C, 2D (Supplementary Table S8), respectively. Further studies about biological functions of these pathways may help us understand the occurrence and development of CTD-ILD.

### Expression Levels of Six Selected Target Genes in Patients with ILD and NILD

**Figure 3:**
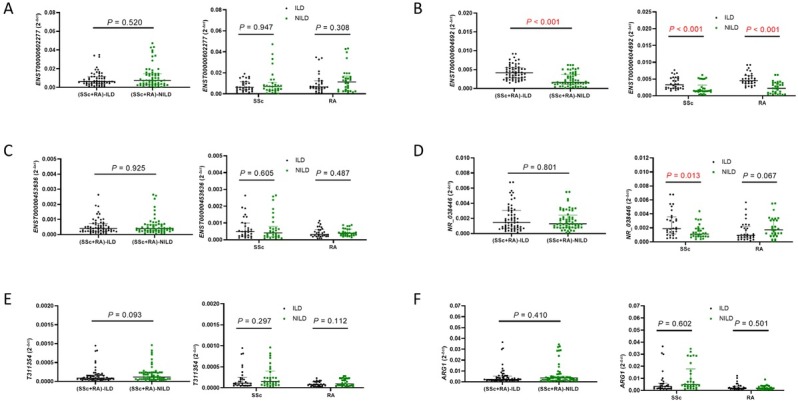
Relative expression (qRT-PCR) of the five lncRNAs and one mRNA in ILD patients (30 SSc-ILD, 30 RA-ILD) and NILD patients (30 SSc-NILD, 30 RA-NILD). ILD, interstitial lung disease; NILD, without interstitial lung disease; SSc, systemic sclerosis; RA, rheumatoid arthritis. P < 0.05 was considered to indicate statistical significance.

The comparison between ILD group and NILD group was shown in Figure 3. *ENST00000604692* was significantly higher in the ILD group than the NILD group, and it was also significantly higher in the SSc-ILD and RA-ILD groups than the SSc-NILD and RA-NILD groups, respectively; *NR_038446* were significantly higher in the SSc-ILD group than the SSc-NILD group. The expression levels of *ENST00000602277*, *ENST00000453636*, *T311354*, and *ARG1* were not significantly diferent between the ILD and NILD groups.

### Expression Levels of Six Selected Target Genes in Patients with SSc and RA

**Figure 4:**
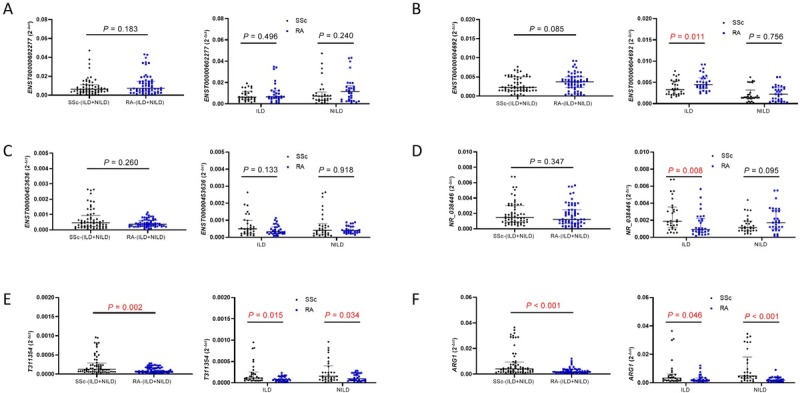
Relative expression (qRT-PCR) of the five lncRNAs and one mRNA in SSc patients (30 patients with SSc-ILD, 30 patients with SSc-NILD) and RA patients (30 patients with RA-ILD, 30 patients with RA-NILD). SSc, systemic sclerosis; RA, rheumatoid arthritis; ILD, interstitial lung disease; NILD, without interstitial lung disease. P < 0.05 was considered to indicate statistical significance.

**Figure 5:**
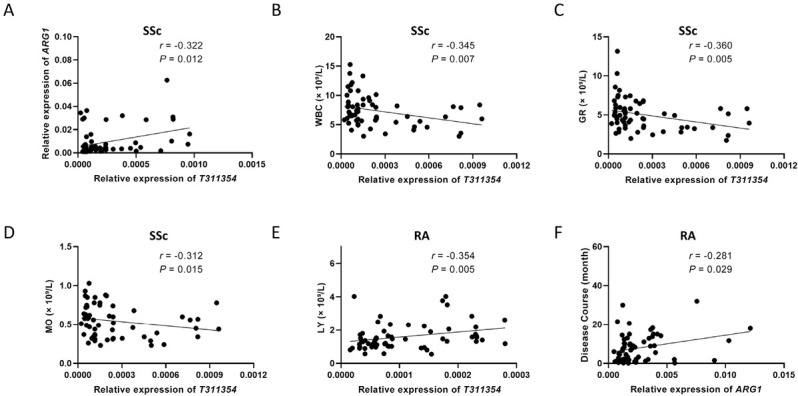
Clinical significance of T311354 and ARG1. (A) The expression level of T311354 in the SSc group was significantly correlated with the expression level of ARG1. (B-D) In the SSc group, the expression level of T311354 was negatively correlated with WBC, GR, and Mo. (E) The expression level of T311354 was positively correlated with LY in the RA group. (F) The expression level of ARG1 was positively correlated with the disease duration in the RA group. SSc, systemic sclerosis; RA, rheumatoid arthritis; WBC, white blood cell counts; GR, neutrophile granulocyte counts; LY, lymphocyte counts; Mo, monocyte counts. P < 0.05 was considered to indicate statistical significance.

The comparison between SSc group and RA group was shown in Figure 4. Expression levels of *T311354* and *ARG1* were both significantly higher in the SSc group than in the RA group. In the ILD patients, *ENST00000604692* level was significantly lower while *NR_038446* level was much higher in the SSc group than that in the RA group. In addition, *ENST00000602277* and *ENST00000453636* showed no diference in the other pairwise group comparisons. Since *ARG1* is a neighboring gene of *T311354*, and qRT-PCR indicated that they were differentially expressed in both the SSc and RA groups, this study analyzed whether there was a correlation between their expression levels in the two groups. As shown in Figure 5A. The expression level of *ARG1* was positively correlated with *T311354* in the SSc group (*r* = 0.322, *P* = 0.012), but not in the RA group.

### Associations of Diferential Target Gene Expression Levels with Laboratory Data

The relative expression of *ENST00000604692* does not correlate with the clinical indicators of the ILD group or the NILD group, respectively. Based on the significant differential expression of *ENST00000604692*, *T311354*, and *ARG1* in the SSc group and the RA group, the correlation between them and the clinical indicators of the SSc group and the RA group was analyzed. In the SSc group, the expression level of *T311354* was negatively correlated with WBC, GR, and Mo (*r* = -0.345, *P* = 0.007; *r* = -0.360, *P* = 0.005; *r* = -0.312, *P* = 0.015). In the RA group, the expression level of *T311354* was positively correlated with LY (*r* = 0.354, *P* = 0.005), and the expression level of *ARG1* was positively correlated with the disease duration (*r* = 0.281, *P* = 0.029) (Figure 5B-F).

### ROC Curve Analysis

**Figure 6:**
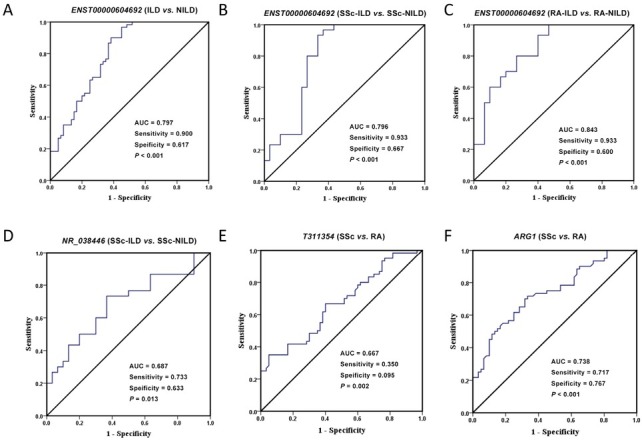
Receiver operating characteristic (ROC) curve analysis of relative expression of diferential target genes. (A) The ability of ENST00000604692 expression level to distinguish between ILD and NILD. (B) The ability of ENST00000604692 expression level to distinguish between SSc-ILD and SSc-NILD. (C) The ability of ENST00000604692 expression level to distinguish between RA-ILD and RA-NILD. (D) The ability of NR_038446 expression level to distinguish between SSc-ILD and SSc-NILD. (E) The ability of T311354 expression level to distinguish between SSc and RA. (F) The ability of ARG1 expression level to distinguish between SSc and RA. AUC, the area under the ROC curve.

The ROC analysis is a valuable statistical tool, which evaluates the sensitivity and the specificity of biomarkers to be used in making a diagnostic decision. Calculate the ROC curve and the area under the curve (AUC) to determine the ability of the differential gene to distinguish the ILD group from the NILD group or the SSc group from the RA group (Figure 6). *ENST00000604692* expression level yielded an AUC value of 0.797 (95% confidence interval [CI]), 0.7170.876; *P* < 0.001; sensitivity = 90.0%; specificity = 61.7%) for discriminating between ILD and NILD. Furthermore, the AUC for *ENST00000604692* in predicting SSc-ILD and RA-ILD was 0.796 (95% CI, 0.675-0.916; *P* < 0.001; sensitivity = 93.3%; specificity = 66.7%) and 0.843 (95% CI, 0.746-0.941; *P* < 0.001; sensitivity = 93.3%; specificity = 60.0%), respectively. The AUC for *NR_038446* in predicting SSc-ILD was 0.687 (95% CI, 0.551-0.822; *P* = 0.013; sensitivity = 73.3%; specificity = 63.3%). The AUC of *T311354* and *ARG1* for discriminating between SSc and RA were 0.667 (95% CI, 0.5030.711; *P* = 0.002; sensitivity = 35.0%; specificity = 95.0%), 0.738 (95% CI, 0.503-0.711; *P* < 0.001; sensitivity = 71.7%; specificity = 76.7%), respectively.

## Discussion

As one type of non-coding RNA, lncRNA can regulate gene expression on multiple levels, such as the epigenetic, transcription, and post-transcription levels.^[4, 5, 6]^ In recent years, an increasing number of studies have found that lncRNA plays an important role in regulating autoimmunity and maintaining immune homeostasis.^[11]^ Moreover, multiple lines of evidence link dysregulation of lncRNA to diverse human autoimmune diseases, such as SSc,^[22]^ RA,^[23]^ SLE,^[24]^ and SS.^[25]^ Previous studies have found that lncRNA is involved in the pathogenesis of SSc-ILD and RA-ILD,^[26,27]^ however, the commonalities and differences amongst CTD-ILD mechanisms are poorly understood. Currently, there are few related studies on lncRNA in CTD-ILD. Thus，elucidating the detailed regulatory mechanism of lncRNA in CTD-ILD is essential for CTD-ILD diagnosis, treatment, and prognostic evaluation. Here we showed the expression profile of lncRNA and mRNA in human CTD-ILD based on microarray analysis. Compared with CTD-NILD patients, there were differentially expressed lncRNA in the PBMCs of CTD-ILD patients. Gaining a better understanding of the regulatory roles of lncRNA in CTD-ILD will provide novel insights into the mechanism of CTD-ILD.

Accumulative evidence indicated possible that genetic, environmental, and immunological factors may participate in the pathogenesis of CTD-ILD.^[2,3]^ In this study, GO and KEGG pathway analyses were performed to predict the biological functions of differentially expressed lncRNA and mRNA in CTD-ILD. In GO analysis, the main function of differential genes was reflected in immune-inflammatory regulation. The KEGG pathway analysis showed that the TLRs/NF-κB signaling pathway was highly enriched. It is speculated that the inflammation mediated by TLRs/NF-κB may contribute to the development of CTD-ILD. Published studies have implicated that TLRs/NF-κB signaling pathway plays a crucial role in the development of certain autoimmune diseases, such as SSc, RA, and SS.^[28, 29, 30]^ In addition, in the KEGG pathway analysis of this study, the differential gene also participated in Chemokine signaling pathway, IL-17 signaling pathway, TNF signaling pathway, transforming growth factor-β (TGF-β) signaling pathway and other signaling pathways related to inflammation regulation. Thus, it may be concluded that both the innate immune system and the adaptive immune system contributed to the development of fibrosis. The study enhances the understanding of specific immune inflammatory response pathways that cause CTD-ILD. Certainly, future research is warranted to address the precise regulatory mechanism of these predicted signal pathways in participating in CTD-ILD.

During disease evolution, patients with SSc, RA, SLE, SS or DM\PM continued to have higher rates of ILD. In the clinical work, SSc-ILD and RA-ILD were the most frequently observed CTD-ILD.^[3]^ Early diagnosis of CTD-ILD is critical. However, there remains a lack of a sensitive, specific, and convenient biomarker to diagnosis the CTD-ILD. Previous studies have shown that lncRNAs have the potential to be novel biomarkers and even therapeutic targets.^[4,5]^ In this study, we speculate that ENST000000604692 may be involved in the pathogenesis of CTD-ILD. ROC curve analysis showed that *ENST00000604692* can efectively distinguish ILD group from NILD group. Combined with subgroup comparison, *ENST00000604692* may be a diagnostic indicator of CTD-ILD, especially SSc-ILD and RA-ILD. Yet, further research is needed to prove this hypothesis. Related research have reported that *NR_038446* may be involved in the pathogenesis of cardiovascular disease, nervous system disease, chronic obstructive pulmonary disease and cancer,^[31, 32, 33, 34]^ while there is no research in CTD-ILD. The relative expression of *NR_038446* was significantly higher in the SSc-ILD group. From this, it was hypothesized that *NR_038446* may not only participate in the pathogenesis of SSc but also participate in the pathogenesis of SSc-ILD. The results described above illustrate that lncRNA has commonalities and diferences in diferent CTD-ILD pathological mechanisms.

ILD, especially CTD-ILD, is characterized by a combination of pro-inflammatory and pro-fibrotic efects. The process involves multiple immune inflammatory cells, especially lung macrophages, which are the most immune cells in the respiratory tract under physiological conditions.^[35]^ According to diferent secreted factors and functions, macrophages can be divided into M1 (activated) and M2 (alternatively activated) phenotypes.^[36]^ The two types antagonize each other and convert each other to some extent，making them play an important role in the progress of CTD-ILD. Previous studies have found that *ARG1* is a biomarker of M2 macrophages.^[37]^ In the macrophages of the lung tissue of idiopathic pulmonary fibrosis patients, the expression of *ARG1* is higher than that of the lung tissue of healthy controls.^[37]^ The microarray results of this study showed that *ARG1* in CTD-ILD group was significantly higher than in CTD group, and analysis of differential lincRNAs and adjacent differential mRNAs (< 300 kb) revealed that *T311354* was correlated with *ARG1*. Moreover, *T311354* had a certain correlation with the immune-inflammatory cells in SSc such as WBC, GR, MO, indicating that *T311354* may be involved in the immune-inflammatory regulation process of SSc. These results prompted us to further study CTD-ILD, especially the molecular mechanism of SSc pathogenesis is very important, but a larger sample or more in-depth mechanism research is necessary.

There are some limitations in the present study to be considered. First, this study has a relatively small sample size. While high throughput behavioral analysis has many advantages, poor repeatability was also inevitable, so it may bring some errors to the screening of differentially expressed genes. Second, the study only carried out comparisons between disease groups and lacked healthy control groups. Finally, further in vitro and in vivo studies are needed to confirm the role of these target genes in CTD-ILD.

In summary, this study reported the expression profile of lncRNA and mRNA in patients with CTD-ILD，which was not found in previous studies. The results provided novel insights into the pathogenesis of CTD-ILD. Additionally, the expression level of *ENST00000604692* was significantly increased in the ILD group, especially SSc-ILD and RA-ILD, which may represent a new biomarker for the diagnosis of CTD-ILD. And *T311354* was more likely to participate in the pathogenesis of SSc or RA by regulating *ARG1*. Of course, the molecular mechanisms and specific functions of these selected target genes in CTD-ILD or CTD need to be further studied.

## Supplementary Material

Supplementary Material Details
