# Supplementary material for: Identification and functional prediction of long non-coding RNA and mRNA related to connective tissue disease-associated interstitial lung diseases
Source: Rheumatol Immunol Res. 2023 Dec 19;4(4):204–15. doi: 10.2478/rir-2023-0030 (PMC10729597; doi:10.2478/rir-2023-0030)
Supplement: Supplementary file 1 — Supplementary Material Details [file rir-2023-0030_sm.pdf]

Supplementary Materials

Table S1: General data of study population for lncRNA microarray analysis

	CTD-ILD group (mixed specimens, *n* = 4)	CTD-NILD group (mixed specimens, *n* = 3)
Age (years)	38.56 ± 13.29	38.58 ± 13.84
M/F (case)	3/13	2/10
SSc, M/F (case)	1/3	1/2
RA, M/F (case)	1/3	1/2
SLE, M/F (case)	0/4	0/3
SS, M/F (case)	1/3	0/3

Values were expressed as numbers or the mean ± standard deviation. CTD-ILD, connective tissue disease-associated interstitial lung diseases; CTD-NILD, connective tissue disease-without interstitial lung diseases; SSc, systemic sclerosis; RA, rheumatoid arthritis; SLE, systemic lupus erythematosus; SS, Sjogren's syndrome; M, male; F, female.

Table S2: Clinical and laboratory data of of SSc and RA

	(SSc+RA)-ILD (*n* = 60)	(SSc+RA)-NILD (*n* = 60)	SSc-(ILD+NILD) (*n* = 60)	RA-(ILD+NILD) (*n* = 60)	SSc-ILD (*n* = 30)	SSc-NILD (*n* = 30)	RA-ILD (*n* = 30)	RA-NILD (*n* = 30)
Age, mean ± SD years	55.18(10.21)	54.00(9.95)	53.98(9.32)	55.20(10.79)	54.57(9.04)	53.4(9.72)	55.8(11.39)	54.6(10.31)
Male/female	15/45	14/46	15/45	14/46	8/22	7/23	7/23	7/23
Disease duration median (IQR) years	6.10(1.90-10.83)[a]	2.25(1.23-6.95)	3.00(1.30-6.15)[b]	6.65(1.58-12.68)	4.50(1.00-7.25)	2.10(1.30-4.48)	7.90(3.30-13.38)	2.50(1.18-11.45)
ESR, mean ± SD (mm/h)	48.80(36.62)	39.27(35.24)	33.92(32.22)[b]	54.15(37.18)	39.73(37.81)	28.1(24.76)	57.87(33.6)	50.43(40.68)
CRP, mean ± SD (mg/L)	19.96(30.28)	14.48(29.22)	8.61(11.87)[b]	25.83(38.66)	11.89(14.07)[c]	5.34(8.14)	28.03(39.15)	23.62(38.7)
WBC, mean ± SD (× 10^9/L)	7.58(3.53)	7.23(2.86)	7.38(2.67)	7.44(3.68)	7.4(2.88)	7.36(2.50)	7.76(4.12)	7.11(3.22)
GR, mean ± SD (× 10^9/L)	5.26(2.89)	4.98(2.55)	5.02(2.06)	5.22(3.27)	5.07(2.30)	4.97(1.82)	5.46(3.42)	4.98(3.15)
LY, mean ± SD (× 10^9/L)	1.61(0.88)	1.66(0.76)	1.66(0.83)	1.60(0.80)	1.62(0.85)	1.71(0.83)	1.6(0.92)	1.6)(0.69)
MO, mean ± SD (× 10^9/L)	0.55(0.26)[a]	0.45(0.18)	0.55(0.21)[b]	0.45(0.23)	0.58(0.21)	0.51(0.20)	0.52(0.3)[d]	0.39(0.13)
ALB, mean ± SD (g/L)	40.77(5.11)[a]	42.7(3.70)	41.1(4.18)	42.37(4.84)	40.37(4.49)	41.82(3.79)	41.16(5.72)	43.57(3.45)
GLOB, mean ± SD (g/L)	31.07(6.47)	32.48(5.86)	33.19(6.91)[b]	30.36(5.04)	32.85(7.47)	33.53(6.4)	29.28(4.77)	31.43(5.15)
Anticentromere antibody	-	-	19(31.67%)	-	10(33.33%)	9(30.00%)	-	-
Anti-topoisomerase I	-	-	8(13.33%)	-	4(13.33%)	4(13.33%)	-	-
Anti-Scl-70	-	-	21(35.00%)	-	11(36.67%)	10(33.33%)	-	-
RF-IgM, median (IQR) (IU/mL)	-	-	-	132.50(33.08-337.25)	-	-	123.5(32.80-285.50)	143.00(33.45-443.00)
RF-IgG, median (IQR) (U/mL)	-	-	-	5.09(3.36-8.20)	-	-	5.02(3.16-6.13)	5.41(3.85-11.66)
RF-IgA, median (IQR) (U/mL)	-	-	-	45.95(22.33-132.77)	-	-	51.73(30.16-127.28)	42.60(10.07-160.13)
CCP, median (IQR) (RU/mL)	-	-	-	313.19(34.19-653.55)	-	-	260.79(49.54-659.00)	105.02(25.50-482.43)

SSc, systemic sclerosis; RA, rheumatoid arthritis; ILD, interstitial lung disease; NILD, without interstitial lung disease; ESR, erythrocyte sedimentation rate; CRP, reactive protein; WBC, white blood cell counts; GR, neutrophile granulocyte counts; LY, lymphocyte counts; Mo, monocyte counts; ALB, albumin; GLOB, globulin; RF, rheumatoid factor; CCP, anti-cyclic citrullinated peptide antibody. Data are presented as the mean with standard deviation (SD), median with interquartile range (IQR), and numbers with percentages. Differences were tested by independent-sample *T*-test, Mann-Whitney *U*-test, and *Chi-square* test. $P < 0.05$ was considered to denote statistical significance, [a]: in comparison with (SSc+RA)-NILD group, [b]: in comparison with RA-(ILD+NILD) group, [c]: in comparison with SSc-NILD group, [d]: in comparison with RA-NILD group.

Table S3: Primer sequences used in the validation of genes

Gene name	Forward primers sequence(5′-3′)	Reverse primers sequence(5′-3′)
β-actin	GAGCTACGAGCTGCCTGACG	GTAGTTTCGTGGATGCCACAG
ENST00000602277	CCTCCTATCAATCTCAGACTGCTGT	CCCCTCCTGAATGAAGACGTCT
ENST00000604692	TCATGCCATCACGGGAAACAT	ACCAACCAAAACCTAAGGAAAACAC
T311354	CCCTGCTTCCAACTGCCTTG	ATGCAGTCACTGTTCCTCTCTTG
ENST00000453636	CTCGCCGAAGTGTCTTGCTGAAG	TTGCCTCTCTGGTTTGTGTTGACTG
NR_038446	ATTCCTACCATGCTACCAACAGACAAG	GTGACTGGCAATACAGGGACCTAAAG
ARG1(arginase-1)	GGACCTGCCCTTTGCTGACATC	AGGCTGATTCTTCCGTTCTTCTTGAC

Table S4: Details of ten pairs of lincRNA-mRNA

lincRNA	mRNA	Chrom
ENST00000492960	ANKRD36	chr2
ENST00000493529	SERPINI1	chr3
ENST00000606452	TIGD5	chr8
NR_024627	SLC22A18AS	chr11
NR_037867	ACSL1	chr4
T140209	ZNF232	chr17
T200956	TNFAIP6	chr2
T260556	S100P	chr4
T311354	ARG1	chr6
TCONS_l2_00009699	RP11-812E19.9	chr16

Chrom, chromosome number from which the lincRNA or mRNA was transcribed.

Table S5: Details of two pairs of antisense lncRNA-mRNA (distance < 300 kb)

Antisense lncRNA	mRNA	Chrom
ENST00000464659	GK	chrX
NR_109857	WDR86	chr7

Chrom, chromosome number from which the lincRNA or mRNA was transcribed.

Table S6: Details of five lncRNAs and one mRNA

Transcript_type	Gene name	*P*-value	Fold change	Regulation	Chrom
long noncoding	ENST00000602277	0.008641533	4.7558537	up	chrX
long noncoding	ENST00000604692	0.033988100	2.6110634	up	chr2
long noncoding	T311354	0.01215425	2.4733744	up	chr6
long noncoding	ENST00000453636	0.002489511	3.7806322	down	chr2
long noncoding	NR_038446	0.046031098	2.6222282	down	chr8
protein_coding	ARG1(arginase-1)	0.019525748	6.9776168	up	chr6

P-value, *P*-value calculated from unpaired *t*-test; Fold change, the absolute ratio (no log scale) of normalized intensities between two groups (CTD-ILD *vs* CTD-NILD); Chrom, chromosome number from which the lncRNA or mRNA was transcribed.

Table S7: Gene Ontology (GO) analysis enriches the top 10 items in the molecular function, cell composition, and biological process of upregulated mRNAs

GO.ID	Term	Gene.Ontology	Count	*P* value	Enrichment. Score(-log10(*P* value))	Genes
GO:0030593	neutrophil chemotaxis	Biological process	13	6.07217×10^{-14}	13.21665624	*CXCL8//THBS4//CXCL1//CXCL2//CXCL3//IL1B//CCL4L1//CCL3L3//CCL3//CCL3L1//CCL18//CCL20//CCL24*
GO:0070098	chemokine-mediated signaling pathway	Biological process	12	2.26516×10^{-13}	12.64490043	*CXCL1//CXCL2//CXCL3//CXCL8//CCL4L1//CCL3L3//CCL3//CCL3L1//CCL18//CCL20//CCL24//CCRL2*
GO:1990266	neutrophil migration	Biological process	13	5.65549×10^{-13}	12.24752981	*CXCL1//CXCL2//CXCL3//IL1B//CXCL8//CCL4L1//CCL3L3//CCL3//CCL3L1//CCL18//CCL20//CCL24//THBS4*
GO:1990868	response to chemokine	Biological process	12	7.50896×10^{-13}	12.12442021	*CXCL1//CXCL2//CXCL3//CXCL8//CCL4L1//CCL3L3//CCL3//CCL3L1//CCL18//CCL20//CCL24//CCRL2*
GO:1990869	cellular response to chemokine	Biological process	12	7.50896×10^{-13}	12.12442021	*CXCL1//CXCL2//CXCL3//CXCL8//CCL4L1//CCL3L3//CCL3//CCL3L1//CCL18//CCL20//CCL24//CCRL2*
GO:0071621	granulocyte chemotaxis	Biological process	13	9.54757×10^{-13}	12.02010724	*CXCL1//CXCL2//CXCL3//IL1B//CXCL8//CCL4L1//CCL3L3//CCL3//CCL3L1//CCL18//CCL20//CCL24//THBS4*
GO:0097530	granulocyte migration	Biological process	13	8.17045×10^{-12}	11.08775416	*CXCL1//CXCL2//CXCL3//IL1B//CXCL8//CCL4L1//CCL3L3//CCL3//CCL3L1//CCL18//CCL20//CCL24//THBS4*
GO:0030595	leukocyte chemotaxis	Biological process	15	1.29557×10^{-11}	10.88753902	*GPR183//CCL4L1//CCL3L3//CCL3//CCL3L1//CCL18//CCL20//CCL24//DEFA1//CXCL1//CXCL2//CXCL3//IL1B//CXCL8//THBS4*
GO:0006950	response to stress	Biological process	58	3.23412×10^{-11}	10.49024433	*CDC45//IL1A//IL1B//NAMPT//DEFA1//LTF//HAVCR2//UCHL1//GADD45A//CDK1//CCNH//WDR33//CRISP3//INHBA//CD83//GBP5//CXCL1//CXCL2//CXCL3//CXCL8//CCL4L1//CCL3L3//NFE2L2//PTGS2//CCL3//CCL3L1//CCL18//CCL20//CCL24//NFKBIZ//TNFAIP6//NFKBID//CCRL2//FOSL1//E2F7//RGCC//E2F8//THBS4//PLAUR//BCL2A1//MELK//METRNL//ATP2B1//ARG1//RNF103//ATF3//FANCI//BIRC3//SERPINB2//ANXA3//AP1G1//CD55//CCNA2//SLC12A6//NUP98//SKIL//PPP1R10//IER3*
GO:0060326	cell chemotaxis	Biological process	16	7.96729×10^{-11}	10.09868951	*GPR183//CCL4L1//CCL3L3//CCL3//CCL3L1//CCL18//CCL20//CCL24//DEFA1//CXCL1//CXCL2//CXCL3//IL1B//CXCL8//THBS4//CCRL2*
GO:0042581	specific granule	Cellular component	8	5.05935×10^{-6}	5.295904849	*PLAUR//SLC2A3//CRISP3//CXCL1//ARG1//LTF//TCN1//ANXA3*
GO:1904724	tertiary granule lumen	Cellular component	5	1.84733×10^{-5}	4.733455402	*CRISP3//CXCL1//LTF//TCN1//TNFAIP6*
GO:0035580	specific granule lumen	Cellular component	5	3.32468×10^{-5}	4.478249933	*CRISP3//CXCL1//ARG1//LTF//TCN1*
GO:0030141	secretory granule	Cellular component	16	4.54057×10^{-5}	4.342889919	*SKIL//ABCA13//CD55//SLC2A3//GPI//S100P//DEFA1//ARG1//PLAUR//CRISP3//CXCL1//LTF//TCN1//ANXA3//TNFAIP6//IL1B*
GO:0070820	tertiary granule	Cellular component	7	5.55607×10^{-5}	4.255232081	*SLC2A3//CD55//CRISP3//CXCL1//LTF//TCN1//TNFAIP6*
GO:0099503	secretory vesicle	Cellular component	16	0.000333796	3.476519221	*SKIL//IL1B//LTF//ABCA13//CD55//SLC2A3//GPI//S100P//DEFA1//ARG1//PLAUR//CRISP3//CXCL1//TCN1//ANXA3//TNFAIP6*
GO:0034774	secretory granule lumen	Cellular component	8	0.000674105	3.171272193	*DEFA1//ARG1//CRISP3//CXCL1//LTF//TCN1//GPI//S100P*
GO:0060205	cytoplasmic vesicle lumen	Cellular component	8	0.000730487	3.136387666	*GPI//S100P//DEFA1//ARG1//CRISP3//CXCL1//LTF//TCN1*
GO:0031983	vesicle lumen	Cellular component	8	0.00076007	3.119146212	*GPI//S100P//DEFA1//ARG1//CRISP3//CXCL1//LTF//TCN1*

GO:0005615	extracellular space	Cellular component	35	0.001528587	2.815709936	*INHBA//NAMPT//CD55//DEFA1//GK//GPI//METRNL//ATAD2//ANXA3//LTF//ATP2B1//ERMN//S100P//SLC2A3//THBS4//CDK1//CRISP3//SCGB3A2//CXCL1//CXCL2//CXCL3//IL1A//IL1B//CXCL8//ARG1//CCL4L1//CCL3L3//SERPINB2//CCL3//CCL3L1//CCL18//CCL20//CCL24//TCN1//TNFAIP6*
GO:0008009	chemokine activity	Molecular function	11	9.43998×10^{-15}	14.0250288	*CXCL1//CXCL2//CXCL3//CXCL8//CCL4L1//CCL3L3//CCL3//CCL3L1//CCL18//CCL20//CCL24*
GO:0042379	chemokine receptor binding	Molecular function	12	1.77742×10^{-14}	13.75020881	*CXCL8//CXCL1//CXCL2//CXCL3//CCL4L1//CCL3L3//CCL3//CCL3L1//CCL18//CCL20//CCL24//CCRL2*
GO:0005125	cytokine activity	Molecular function	16	1.74962×10^{-12}	11.75705636	*CXCL1//CXCL2//CXCL3//CXCL8//CCL4L1//CCL3L3//CCL3//CCL3L1//CCL18//CCL20//CCL24//NAMPT//GPI//IL1A//IL1B//INHBA*
GO:0048020	CCR chemokine receptor binding	Molecular function	8	5.10992×10^{-10}	9.2915855	*CCL3//CCL24//CCL20//CCL4L1//CCL3L3//CCL3L1//CCL18//CCRL2*
GO:0048018	receptor ligand activity	Molecular function	18	1.59097×10^{-9}	8.798337049	*NAMPT//GPI//IL1A//IL1B//INHBA//METRNL//CXCL1//CXCL2//CXCL3//CXCL8//CCL4L1//CCL3L3//CCL3//CCL3L1//CCL18//CCL20//CCL24//THBS4*
GO:0005126	cytokine receptor binding	Molecular function	14	1.77089×10^{-9}	8.75180935	*IL1A//IL1B//CXCL8//CXCL1//CXCL2//CXCL3//CCL4L1//CCL3L3//CCL3//CCL3L1//CCL18//CCL20//CCL24//CCRL2*
GO:0030546	signaling receptor activator activity	Molecular function	18	1.86949×10^{-9}	8.728277929	*NAMPT//GPI//IL1A//IL1B//INHBA//METRNL//CXCL1//CXCL2//CXCL3//CXCL8//CCL4L1//CCL3L3//CCL3//CCL3L1//CCL18//CCL20//CCL24//THBS4*
GO:0030545	receptor regulator activity	Molecular function	18	6.74076×10^{-9}	8.171291332	*NAMPT//GPI//IL1A//IL1B//INHBA//METRNL//CXCL1//CXCL2//CXCL3//CXCL8//CCL4L1//CCL3L3//CCL3//CCL3L1//CCL18//CCL20//CCL24//THBS4*
GO:0001664	G protein-coupled receptor binding	Molecular function	13	4.25292×10^{-8}	7.371312408	*CXCL8//CXCL1//CXCL2//CXCL3//CCL4L1//CCL3L3//CCL3//CCL3L1//CCL18//CCL20//CCL24//UCHL1//CCRL2*
GO:0098772	molecular function regulator	Molecular function	46	2.27062×10^{-6}	5.643855999	*E2F7//ATF3//NFE2L2//SKIL//TBX15//E2F8//FOSL1//KLF7//ZNF267//ZNF644//MYSM1//NUP98//GMNN//PHACTR1//PPP1R10//SERPINB2//LTF//FGD6//RASGEF1B//DEPDC1B//NAMPT//GPI//IL1A//IL1B//INHBA//METRNL//CXCL1//CXCL2//CXCL3//CXCL8//CCL4L1//CCL3L3//CCL3//CCL3L1//CCL18//CCL20//CCL24//THBS4//ST20//CCNH//CCNA2//DENND3//ANXA3//RGCC//BIRC3//SOCS4*

Table S7: Gene Ontology (GO) analysis enriches the top 10 items in the molecular function, cell composition, and biological process of downregulated mRNAs

GO.ID	Term	Gene.Ontology	Count	*P* value	Enrichment. Score (-log10 (*P* value))	Genes
GO:0050854	regulation of antigen receptor-mediated signaling pathway	Biological process	3	0.001663014	2.779104055	*CD19//PAWR//STAP1*
GO:1901880	negative regulation of protein depolymerization	Biological process	3	0.001805414	2.743423243	*CAMSAP2//AVIL//ADD1*
GO:0046058	cAMP metabolic process	Biological process	2	0.002504874	2.60121414	*ADCY9//PDE7A*
GO:0043242	negative regulation of protein-containing complex disassembly	Biological process	3	0.002631008	2.579877824	*CAMSAP2//AVIL//ADD1*
GO:0046339	diacylglycerol metabolic process	Biological process	2	0.003231852	2.490548567	*GPAM//AVIL*

GO:1901879	regulation of protein depolymerization	Biological process	3	0.003327459	2.477887293	*CAMSAP2//AVIL//ADD1*
GO:0120032	regulation of plasma membrane bounded cell projection assembly	Biological process	4	0.003459571	2.460977741	*AVIL//C15ORF62//STAP1//EPS8L1*
GO:0051016	barbed-end actin filament capping	Biological process	2	0.003493543	2.456733907	*AVIL//ADD1*
GO:0060491	regulation of cell projection assembly	Biological process	4	0.003596459	2.444124875	*AVIL//C15ORF62//STAP1//EPS8L1*
GO:0050855	regulation of B cell receptor signaling pathway	Biological process	2	0.004335786	2.362932206	*STAP1//CD19*
GO:0015629	actin cytoskeleton	Cellular component	7	0.002010957	2.696597316	*SIPA1L3//ACTL7B//AVIL//PAWR//ADD1//GYS2//DDX58*
GO:0031256	leading edge membrane	Cellular component	4	0.00340537	2.46783565	*CNTNAP2//DDX58//EPS8L1//SGCE*
GO:0005911	cell-cell junction	Cellular component	6	0.007811412	2.107270449	*ADD1//TNKS1BP1//DDX58//MAGI2//CNTNAP2//SIPA1L3*
GO:0070160	tight junction	Cellular component	3	0.010700046	1.970614347	*DDX58//MAGI2//SIPA1L3*
GO:0005887	integral component of plasma membrane	Cellular component	12	0.012156394	1.915195247	*CNTNAP2//KCNG1//ADCY9//FCRL1//DCBLD2//ADORA3//OPN3//SLC10A1//TLR5//FCRLA//SGCE//CD19*
GO:0031252	cell leading edge	Cellular component	5	0.016451279	1.783800323	*AVIL//CNTNAP2//DDX58//EPS8L1//SGCE*
GO:0031226	intrinsic component of plasma membrane	Cellular component	12	0.017172376	1.765169621	*ADCY9//FCRL1//DCBLD2//ADORA3//OPN3//SLC10A1//TLR5//FCRLA//SGCE//CD19//CNTNAP2//KCNG1*
GO:0000790	nuclear chromatin	Cellular component	10	0.018177597	1.74046353	*PPARGC1A//TNKS1BP1//E2F5//ZNF718//SMAD9//ARGFX//PAWR//NEUROG2//ZNF232//ZNF697*
GO:0032589	neuron projection membrane	Cellular component	2	0.019632547	1.707023363	*CNTNAP2//SGCE*
GO:0043005	neuron projection	Cellular component	10	0.021080223	1.676124805	*OPN3//PDC//AVIL//ADCY9//CNTNAP2//PIAS3//MAGI2//CDK5R2//SGCE//PPARGC1A*
GO:0030159	signaling receptor complex adaptor activity	Molecular function	3	0.000510617	3.291904622	*STAP1//BLNK//MAGI2*
GO:0005068	transmembrane receptor protein tyrosine kinase adaptor activity	Molecular function	2	0.001088674	2.96310215	*STAP1//BLNK*
GO:0035591	signaling adaptor activity	Molecular function	3	0.002230455	2.651606456	*STAP1//BLNK//MAGI2*
GO:0030507	spectrin binding	Molecular function	2	0.005084637	2.293740017	*ADD1//CAMSAP2*
GO:0016779	nucleotidyltransferase activity	Molecular function	3	0.014310479	1.844345816	*NMNAT1//POLR1A//CTU1*
GO:0005089	Rho guanyl-nucleotide exchange factor activity	Molecular function	2	0.022016884	1.657244146	*EPS8L1//PLEKHG1*
GO:0017048	Rho GTPase binding	Molecular function	3	0.024023052	1.619371813	*EPS8L1//PLEKHG1//C15ORF62*
GO:0003779	actin binding	Molecular function	5	0.025597474	1.591802885	*AVIL//ADD1//PAWR//EPS8L1//CDK5R2*
GO:0030971	receptor tyrosine kinase binding	Molecular function	2	0.030880058	1.510321888	*STAP1//BLNK*
GO:0001618	virus receptor activity	Molecular function	2	0.032473957	1.488464789	*CR2//SLC10A1*

Table S8: Kyoto encyclopedia of genes and genomes (KEGG) analysis enriched the top 10 items of upregulated mRNAs

PathwayID	Definition	OriginalWebSite	Fisher-*P* value	Selection-Counts	Enrichment_Score	Genes
hsa04061	Viral protein interaction with cytokine and cytokine receptor - Homo sapiens (human)	http://www.genome.jp/kegg-bin/show_pathway?hsa04061+6362+6364+6369+6348+6349+414062-+388372+9560+2919+2920+2921+3576	7.04606×10^{-12}	12	11.152053	*CCL18//CCL20//CCL24//CCL3//CCL3L1//CCL3L3//CCL4L1//CCL4L2//CXCL1//CXCL2//CXCL3//CXCL8*
hsa04064	NF-kappa B signaling pathway - Homo sapiens (human)	http://www.genome.jp/kegg-bin/show_pathway?hsa04064+597+330+388372+9560+2919+2920+2921+3576+1647+3553+5743	2.32827×10^{-10}	11	9.632967	*BCL2A1//BIRC3//CCL4L1//CCL4L2//CXCL1//CXCL2//CXCL3//CXCL8//GADD45A//IL1B//PTGS2*
hsa05323	Rheumatoid arthritis - Homo sapiens (human)	http://www.genome.jp/kegg-bin/show_pathway?hsa05323+6364+6348+6349+414062+2919+2920+2921+3576+3552+3553	1.4042×10^{-9}	10	8.852571	*CCL20//CCL3//CCL3L1//CCL3L3//CXCL1//CXCL2//CXCL3//CXCL8//IL1A//IL1B*
hsa04060	Cytokine-cytokine receptor interaction - Homo sapiens (human)	http://www.genome.jp/kegg-bin/show_pathway?hsa04060+6362+6364+6369+6348+6349+414062+388372+9560+2919+2920+2921+3576+3552+3553+3624	3.01729×10^{-9}	15	8.520384	*CCL18//CCL20//CCL24//CCL3//CCL3L1//CCL3L3//CCL4L1//CCL4L2//CXCL1//CXCL2//CXCL3//CXCL8//IL1A//IL1B//INHBA*
hsa04062	Chemokine signaling pathway - Homo sapiens (human)	http://www.genome.jp/kegg-bin/show_pathway?hsa04062+6362+6364+6369+6348+6349+414062+388372+9560+2919+2920+2921+3576	1.46616×10^{-8}	12	7.83382	*CCL18//CCL20//CCL24//CCL3//CCL3L1//CCL3L3//CCL4L1//CCL4L2//CXCL1//CXCL2//CXCL3//CXCL8*
hsa04657	IL-17 signaling pathway - Homo sapiens (human)	http://www.genome.jp/kegg-bin/show_pathway?hsa04657+6364+2919+2920+2921+3576+8061+3553+5743	4.45069×10^{-7}	8	6.351573	*CCL20//CXCL1//CXCL2//CXCL3//CXCL8//FOSL1//IL1B//PTGS2*
hsa04621	NOD-like receptor signaling pathway - Homo sapiens (human)	http://www.genome.jp/kegg-bin/show_pathway?hsa04621+330+2919+2920+2921+3576+1667+115362+3553+10135	7.47666×10^{-6}	9	5.126292	*BIRC3//CXCL1//CXCL2//CXCL3//CXCL8//DEFA1//GBP5//IL1B//NAMPT*
hsa05146	Amoebiasis - Homo sapiens (human)	http://www.genome.jp/kegg-bin/show_pathway?hsa05146+383+2919+2920+2921+3576+3553+3914	1.04464×10^{-5}	7	4.981033	*ARG1//CXCL1//CXCL2//CXCL3//CXCL8//IL1B//LAMB3*
hsa04620	Toll-like receptor signaling pathway - Homo sapiens (human)	http://www.genome.jp/kegg-bin/show_pathway?hsa04620+6348+6349+414062+388372+9560+3576+3553	1.18764×10^{-5}	7	4.925315	*CCL3//CCL3L1//CCL3L3//CCL4L1//CCL4L2//CXCL8//IL1B*
hsa04668	TNF signaling pathway - Homo sapiens (human)	http://www.genome.jp/kegg-bin/show_pathway?hsa04668+330+6364+2919+2920+2921+3553+5743	1.93226×10^{-5}	7	4.713935	*BIRC3//CCL20//CXCL1//CXCL2//CXCL3//IL1B//PTGS2*

Table S8: Kyoto encyclopedia of genes and genomes (KEGG) analysis enriched the top 9 items (only 9 items in total) of downregulated mRNAs

PathwayID	Definition	OriginalWebSite	Fisher-*P* value	Selection-Counts	Enrichment_Score	Genes
hsa04662	B cell receptor signaling pathway - Homo sapiens (human)	http://www.genome.jp/kegg-bin/show_pathway?hsa04662+29760+930+1380	0.00275818	3	2.559377	*BLNK//CD19//CR2*

hsa05169	Epstein-Barr virus infection - Homo sapiens (human)	http://www.genome.jp/kegg-bin/show_pathway?hsa05169+29760+930+1380+23586	0.004870649	4	2.312413	*BLNK//CD19//CR2//DDX58*
hsa05340	Primary immuno-deficiency - Homo sapiens (human)	http://www.genome.jp/kegg-bin/show_pathway?hsa05340+29760+930	0.007555506	2	2.121736	*BLNK//CD19*
hsa04015	Rap1 signaling pathway - Homo sapiens (human)	http://www.genome.jp/kegg-bin/show_pathway?hsa04015+115+9863+23094	0.03528105	3	1.452458	*ADCY9//MAGI2//SIPA1L3*
hsa04211	Longevity regulat-ing pathway - Homo sapiens (human)	http://www.genome.jp/kegg-bin/show_pathway?hsa04211+115+10891	0.03776135	2	1.422952	*ADCY9//PPARGC1A*
hsa04976	Bile secretion - Homo sapiens (human)	http://www.genome.jp/kegg-bin/show_pathway?hsa04976+115+6554	0.03853793	2	1.414112	*ADCY9//SLC10A1*
hsa05032	Morphine addiction - Homo sapiens (human)	http://www.genome.jp/kegg-bin/show_pathway?hsa05032+115+5150	0.03932068	2	1.405379	*ADCY9//PDE7A*
hsa04350	TGF-beta signaling pathway - Homo sapiens (human)	http://www.genome.jp/kegg-bin/show_pathway?hsa04350+1875+4093	0.04170544	2	1.379807	*E2F5//SMAD9*
hsa04640	Hematopoietic cell lineage - Homo sapiens (human)	http://www.genome.jp/kegg-bin/show_pathway?hsa04640+930+1380	0.04579888	2	1.339145	*CD19//CR2*
